# Discovery of a novel, liver-targeted thyroid hormone receptor-β agonist, CS271011, in the treatment of lipid metabolism disorders

**DOI:** 10.3389/fendo.2023.1109615

**Published:** 2023-01-20

**Authors:** Suwen Lin, Shengjian Huang, Zhou Deng, Yu Zhang, Lin Huang, Yanyi Wu, Shuyan Lv, Zhiyi Wang, Ning Huang, Lan Wang, Ziqi Chen, Guangyin Yu, Weihua Yin, You Zhou, Zhengyu Fang

**Affiliations:** ^1^ Clinical Research Institute, Shenzhen Peking University - The Hong Kong University of Science and Technology Medical Center, Shenzhen, Guangdong, China; ^2^ Early Research & Development Centers, Shenzhen Chipscreen Biosciences Co., Ltd., Shenzhen, Guangdong, China; ^3^ Chengdu Chipscreen Pharmaceutical Ltd., Chengdu, Sichuan, China; ^4^ School of Chemical Engineering and Technology, Hainan University, Haikou, Hainan, China; ^5^ Department of Pathology, Shenzhen Hospital, Peking University, Shenzhen, Guangdong, China

**Keywords:** thyroid hormone receptor, dyslipidaemia, steatosis, triglycerides, Resmetirom

## Abstract

**Introduction:**

Thyroid hormone receptor β (THR-β) plays a critical role in metabolism regulation and has become an attractive target for treating lipid metabolism disorders in recent years. Thus, in this study, we discovered CS271011, a novel THR-β agonist, and assessed the safety and efficiency of CS271011 compared to MGL-3196 *in vitro* and *in vivo*.

**Methods:**

We conducted luciferase reporter gene assays to assess the activation of THR-β and α in vitro. C57BL/6J mice were fed a high-fat diet for 12 weeks, CS271011 was administered by gavage at the dose of 1 mg/kg and 3 mg/kg, and MGL-3196 was administered at the dose of 3 mg/kg for 10 weeks. Body weight, food intake, serum and hepatic parameters, histological analysis, pharmacokinetic studies, RNA sequencing of the liver and heart, and expression of hepatic lipid-metabolic genes were determined to evaluate the safety and efficiency of CS271011.

**Results:**

Compared with MGL-3196, CS271011 showed higher THR-β activation in vitro. In the diet-induced obesity mice model, CS271011 demonstrated favourable pharmacokinetic properties in mice and was enriched in the liver. Finally, CS271011 improved dyslipidaemia and reduced liver steatosis in the diet-induced obesity murine model. Mechanistically, CS271011 and MGL-3196 showed potent regulation of lipid metabolism-related genes.

**Conclusions:**

CS271011 is a potent and liver-targeted THR-β agonist for treating lipid metabolism disorders.

## 1 Introduction

Lipid metabolism disorders have rapidly emerged as a common social health problem and carry a heavy economic burden worldwide. The dysregulation of lipid metabolism contributes to hyperlipidaemia, non-alcoholic fatty liver disease (NAFLD), diabetes, cardiovascular disease (CVD), and cancers (1, 2). Lifestyle change and drug intervention are effective therapeutic options for the disease (3, 4). However, there are still unmet clinical needs to improve the efficacy and safety of current therapies *via* new biological mechanisms.

Thyroid hormones (THs) are hormones produced by the thyroid gland and play essential roles in metabolism and development (5, 6). THs trigger thyroid hormone receptors in the cell to activate signaling of the genome for the regulation of gene expression. Two different thyroid hormone receptors are known, namely, isoform α (THR-α) and β (THR-β), in which the former is predominantly expressed in the heart, bone, intestine, and brain, and the latter is highly expressed in skeletal muscle, kidney, thyroid, and liver (7). When binding to THR-β in the liver, THs demonstrate favourable regulation of lipid metabolism and cholesterol homeostasis. However, overexpression of THR-α in the heart and bones causes toxic effects such as arrhythmia and hypercalcaemia (8). Therefore, discovering selective THR-β agonists is a novel therapeutic strategy for treating metabolic disorders.

In recent years, thyroid hormone analogues have been developed to improve THR-β selectivity and to validate hyperlipidaemia and non-alcoholic steatohepatitis (NASH) (9, 10). In preclinical studies, liver-targeted THR-β agonists such as GC-1 (Sobetirome), VK2809 (MB07811), and KB-141 effectively lower serum lipid levels and reduce liver steatosis in rodent models (11, 12). Unfortunately, most clinical trials of THR-β agonists were terminated at different stages, probably due to bone toxicities through activating THR-α or liver toxicity, hampering the development of novel entities (13, 14). Recently, Madrigal Pharmaceuticals developed Resmetirom (MGL-3196), a liver-targeted, orally active, and selective THR-β agonist (with ~28-fold selectivity for THR-β vs. THR-α) that exhibited surprising efficacy in both preclinical and clinical studies (15). In a phase II clinical trial, during a 36-week treatment period, MGL-3196 significantly reduced the average hepatic lipid levels in patients with NASH (15, 16), and it is now in a phase III clinical trial (17), indicating a promising therapy for the disease.

CS271011 was designed and synthesized by Chipscreen Biosciences and has higher THR-β activity than that of MGL-3196. In this study, we evaluated the safety and potential therapeutic effects of CS271011 on a diet-induced obesity (DIO) rodent model. We showed that CS271011 had equivalent safety, efficacy, and mechanistic profiles to MGL-3196, suggesting that CS271011 is a potent candidate for treating metabolic disorders.

## 2 Materials and methods

### 2.1 Materials

CS271011 was designed and synthesized at Chipscreen Biosciences (Shenzhen, Guangdong, China). The purity of CS271011 was over 99%. Resmetirom (MGL-3196) was purchased from MedChemExpress (Hycultec GmbH, Beutelsbach, Germany). Compounds were dissolved and suspended in sterile DMSO and 0.2% CMC-Na for *in vitro* and *in vivo* administration, respectively. The chow diet was supplied by Guangdong Medical Laboratory Animal Center (Foshan, Guangdong, China), and the high-fat diet (D12492i) was provided by SYSE Biotech Co., Ltd. (Changzhou, Jiangsu, China). Cholesterol (TC), alanine transaminase (ALT), aspartate transaminase (AST), alkaline phosphatase (ALP), and total bilirubin (TBIL) diagnostic kits were purchased from the Jiancheng Bioengineering Institute of Nanjing (Nanjing, Jiangsu, China). The Triglyceride (TG) Colorimetric Assay Kit was purchased from Elabscience (Wuhan, Hubei, China). All primers were synthesized by Genewiz (Suzhou, Jiangsu, China).

### 2.2 Luciferase reporter gene assays

A luciferase reporter gene assay was applied to assess the capacity of drugs to bind and activate thyroid hormone receptor α or thyroid hormone receptor β. For each well, 5x103 CV-1 cells were seeded into 96-well plates and cultured in Dulbecco’s Modified Eagle Medium (DMEM; Hyclone, SH30022) containing 10% fetal bovine serum (FBS; Gibco, 16000–044) and 1% penicillin−streptomycin (P/S; Corning, 30-002-CI) for 24 hours at 37°C and 5% CO2. An expression plasmid mixture of THR-α or THR-β expression plasmids was transiently transfected into liposomes with a firefly luciferase reporter (2:2:1 thyroid hormone receptor α:TRE : GFP or 3:3:1 thyroid hormone receptor β:TRE : GFP) and mixed with the X-tremeGENE HP DNA Transfection Reagent (Roche, Basel, BS, Switzerland). The two different mixtures indicated above were added to the cells and incubated for 24 hours. Then, CS271011 (0.01-30 M), MGL-3196 (0.01-30 M), and T3 (0.000001-1 M) were diluted in DMSO and added to the transfected cells for 22 hours. Next, the medium was withdrawn, and 60 μl of 1X Cell Culture Lysis and 5X reagent (Promega, E153A) was added to each well for lysis. Subsequently, 50 μl of lysate was transferred into a 96-well white plate and measured on a Tecan microplate reader (Männedorf, Switzerland) to determine the GFP fluorescence value (Ex485/Em520). Finally, 30 μl of Luciferase Assay Buffer (Promega, E151A) was immediately added to each well to detect Luci values for 50% activation (AC50) calculation.

### 2.3 DIO mouse study

All animal experiments were performed at Chipscreen Biosciences (Shenzhen, China) according to the National Research Council’s Guide for the Care and Use of Laboratory Animals and the Regulations of Experimental Animal Administration issued by the State Committee of Science and Technology of China and approved by Shenzhen Peking University - The Hong Kong University of Science and Technology Medical Center’s Ethics Committee for the Welfare of Laboratory Animals (approval no. 2022-988). Forty C57BL/6J mice (4-5 weeks, male) were received and raised under a standard 12-hour light/dark cycle (lights off at 7:00 pm). Thirty-five mice were fed a fat-rich diet (SYSE Bio-tech, D12492i; 60% of kcal from fat; Changzhou, China), and the other five mice were fed a control diet (Guangdong Medical Laboratory Animal Center, Foshan, China) ad libitum for 12 weeks. Except for one mouse that died unexpectedly during the model construction, the other mice were randomized and divided into five groups at the beginning of the intervention: Group 1, chow diet control (n=5); Group 2, DIO control (n=8); Group 3, CS271011 1 mg/kg (n=9); Group 4, CS271011 3 mg/kg (n=9); and Group 5, MGL-3196 3 mg/kg (n=8). The mice from Group 2 to Group 5 were fed a high-fat diet. Treatments started on the thirteenth week and lasted 10 weeks, in which Groups 1 and 2 received solvent (0.2% CMC-Na), and the other groups were administered corresponding compounds orally once daily (the volume of solvent was 5 ml/kg). Body weight and food intake were measured once daily during treatment. All mice were sacrificed after treatment, and blood, hearts, and livers were collected for further validation. In addition, the dosage selection of CS271011 was based on the results of the luciferase reporter gene assay of THR-β and the experiment with MGL-3196 (3 mg/kg) applied in the DIO mouse model and DIO-NASH mouse model (15, 18).

### 2.4 Pharmacokinetic studies

Eighteen C57BL/6J mice (5-6 weeks, male) were randomly divided into two groups and treated with CS271011 3 mg/kg and MGL-3196 3 mg/kg once by gavage, and the volume of solvent was 5 ml/kg. Then, each group was subdivided into three groups for blood extraction: Group 1, sample collection after 0.5 hours and 2 hours of treatment (n=3); Group 2, sample collection after 1 hour and 4 hours of treatment (n=3); and Group 3, sample collection after 8 hours and 24 hours of treatment (n=3). Approximately 100 μl of blood was collected from the orbit and centrifuged (3500 g, 15 min, 4°C) to extract serum and placed on ice (0°C), and 20 μl of serum was added to 200 μl of extractant (containing 200 ng/ml methanol), which was assessed by liquid chromatography-tandem mass spectrometry (LC−MS/MS) procedures.

To detect the tissue distribution of drugs, the heart, liver, and kidney of mice in Group 1, Group 2, and Group 3 were collected at 2 hours, 4 hours, and 24 hours, respectively. Then, 1 ml of 1X PBS was added for tissue homogenization. 50 μl tissue homogenate was added to 500 μl of extractant (containing 200 ng/ml methanol). The protein of tissue samples was precipitated by vortex oscillation for 5 min and centrifuged at 13,000 rpm for 10 min, and 150 μL of supernatant was collected and determined by LC−MS/MS procedures. All the pharmacokinetic (PK) parameters of CS271011 and MGL-3196 were calculated by Excel 2019.

### 2.5 Analysis of serum and liver biochemical indices

Blood samples were collected from the orbit and placed for 30 min at room temperature and then centrifuged (3500 g, 15 min, 4°C), and the serum was collected for TG, TC, AST, and ALT tests. For the liver TG and TC tests, the right lobe of the liver was collected and weighed accurately, and then isopropanol and absolute ethanol were added at a ratio of 1:9 (weight/volume) for tissue homogenization on ice. Next, the liver sample was centrifuged (10,000 g and 2500 g, 10 min, at 4°C for liver TG and TC extraction, respectively), and the supernatant was collected for liver TG and TC assays. Tests for TC, ALT and AST were performed following the manufacturer’s instructions.

### 2.6 Histological analyses

After the animals were sacrificed, the left hepatic lobe was collected (approximately 100–200 mg) and fixed in 4% paraformaldehyde overnight. The liver tissue was sectioned at a thickness of 5 μm after being paraffin-embedded. Liver sections were stained with haematoxylin and eosin (H&E), and the NAFLD activity scores were determined based on the steatosis, lobular inflammation, and hepatocyte ballooning degeneration in accordance with the standard proposed by Kleiner (17).

To analyse the content of lipids in the liver, Oil Red O (ORO) staining of isobutyl alcohol-frozen liver sections was conducted by Wuhan Servicebio Technology Co., Ltd. (Wuhan, China). Each positive region of ORO staining was quantified for each sample using Imangepro plus 6.0 software (National Institute of Mental Health, USA).

### 2.7 RNA sequencing

The right hepatic lobe and heart tissues were randomly collected from three mice per group, stabilized overnight in RNAlater^®^ solution (Melunbio, Dalian, Liaoning, China), and stored at -80°C. RNA-seq was screened and analyzed by Tianjin Novogene Bioinformatic Technology Co., Ltd. (Tianjin, China). Volcano plot, GO, and KEGG enrichment analysis was performed on R software (version 4.2.1), and the UpSet plot was plotted by BioLadder (https://www.bioladder.cn/). Additionally, STRING (https://cn.string-db.org/) and Cytoscape (version v3.9.1) software were employed to study the protein−protein interaction (PPI) network, and the Gene Set Enrichment Analysis of DEGs was performed by using GSEA software (version 4.2.3).

### 2.8 RNA extraction and RT−PCR

The total RNA of fresh liver tissue was extracted using TRIzol reagent (Invitrogen, Carlsbad, CA, USA), and RNA quality was measured using a Nanodrop2000 by calculating the 260/280 ratio. Based on the manufacturer’s instructions, we reverse-transcribed 5 μg of isolated RNA with a Transcriptor First Strand cDNA Synthesis Kit (Roche Diagnostics, Mannheim, Germany). Real-time PCR (RT−PCR) was conducted to measure the relative expression of genes by using SYBR Green Master (ROX) dye (Roche Diagnostics, Mannheim, Germany) and the ABI Prism 7000 Sequence Detection System (Applied Biosystems, Foster City, CA, USA). Changes in target mRNA were standardized to β-actin mRNA levels for each sample in triplicate. The 2-^△△^Ct method was used to determine the relative mRNA expression levels. **Supplementary Table 1** lists the primer sequences utilized in this investigation.

### 2.9 Data and statistical analyses

GraphPad Prism 8 (GraphPad Software, La Jolla, CA, USA) was applied to analyse all data in this study, and the data are represented as the mean ± standard deviation (SD). A 2-tailed Student’s t test compared significant differences between two groups, and one-way ANOVA was performed in three or multiple comparison analyses. *P* values were considered significant at *p <*0.05.

## 3 Results

### 3.1 CS271011 shows high thyroid hormone receptor β activity and selectivity

**Figure 1:**
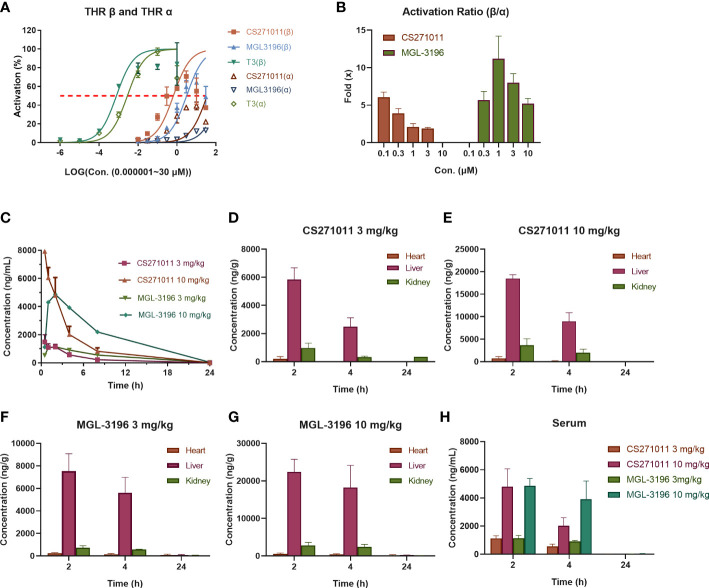
Selective activation of thyroid hormone receptors β and α and pharmacokinetics (PK) of CS271011 and MGL-3196 (n=3 for each subgroup). **(A)** Activation of thyroid hormone receptor β and α; **(B)** Selective activation ratio of thyroid hormone receptor β over thyroid hormone receptor α; **(C)** Serum pharmacokinetic curve of normal mice treated with CS271011 (3 mg/kg and 10 mg/kg) and MGL-3196 (3 mg/kg and 10 mg/kg) orally once. Tissue distribution studies of CS271011 3 mg/kg **(D)**, CS271011 10 mg/kg **(E)**, MGL-3196 3 mg/kg **(F)** and MGL-3196 10 mg/kg **(H)**; **(G)** The serum concentration change of CS271011 and MGL-3196 with different dosages. The red dashed line of **(A, B)** indicates 50% activation of thyroid hormone receptor isoforms. All data are presented as the mean ± SD. T3, Triiodothyronine.

MGL-3196 was reported to have a 28-fold selectivity for THR-β over THR-α in a cell-free coactivator recruitment assay (13). To identify the potency and selectivity of CS271011, we conducted a cell-based luciferase reporter gene assay for both targets. The results showed that CS271011 reached the 50% activation (AC_50_) of THR-α and THR-β with concentrations of 35.17 μM and 0.65 μM, respectively, whereas the result for MGL-3196 was 149.0 μM and 3.11 μM (**Table 1**). Therefore, the activation ratios of THR-α and THR-β for CS271011 and MGL-3196 were 54.12 and 47.93, indicating that CS271011 showed higher THR-β activity over MGL-3196. Both CS271011 and MGL-3196 showed higher THR-β selectivity over T3, a negative control with robust potency in both isoforms but non-pronounced selectivity (**Figure 1A**). CS271011 also exhibited higher THR-α activity than MGL-3196, so we needed to calculate the safety window for both compounds. In addition, both drugs had a similar activity ascent interval, and CS271011 had stronger THRβ activity (**Figure 1B**).

**Table 1:** Activation of THR-β and THR-α for CS271011, MGL-3196 and T3.

Compund		CS271011	MGL-3196	T3
**AC_50_ (μM)**	**β**	0.6499	3.109	0.0007702
	**α**	14.56	149	0.002817
**Selectivity (α/β)**		54.12	47.93	3.66

AC_50_, 50% activation capability.

### 3.2 CS271011 exhibits favorable PK properties and liver-targeted

We then studied the PK properties in normal C57BL/6J mice of a single administration of CS271011 and MGL-3196 at 3 mg/kg and 10 mg/kg by oral gavage (**Table 2**). CS271011 exhibited favorable PK properties, including area under the curve (AUC), mean residence time (MRT), half-life (t_1/2_), peak time (T_max_), and peak concentration (C_max_), in a dose-dependent fashion. The results showed that both doses of CS271011 highly accumulated in the liver than in other tissues, especially 2 and 4 hours after administration (**Figures 1C–E, H**). The liver/serum ratio of CS271011 was approximately 4:1 in both administrations, and the main ratio of liver/serum was approximately 26.2-fold and 5.96-fold higher than that of the heart and kidney, respectively (**Supplementary Table 2**). MGL-3196 also demonstrated favourable PK properties and liver-targeted (**Figures 1C, F–H**) and presented higher bioavailability *in vivo* than CS271011 at the same dosage.

**Table 2:** Pharmacokinetic study in normal C57BL/6J mice by orally once.

Pharmacokinetic index		CS271011(3mg/kg)	MGL-3196 (3mg/kg)	CS271011 (10mg/kg)	MGL-3196 (10mg/kg)
Parameter	Unit				
**AUC_(0-t)_**	**h·ng/mL**	6171.5	9333.9	26002.2	35627.5
**AUC_(0-∞)_**	**h·ng/mL**	6198.5	9472.7	26080.6	35810.1
**MRT_(0-t)_**	**h**	4.3	6.3	3.9	5.8
**t_1/2_**	**h**	3.1	3.8	2.9	3.0
**T_max_**	**h**	0.5	2.0	0.5	2.0
**C_max_**	**ng/mL**	1490.5	1142.5	7929.2	4872.7

AUC, area under curve; MRT, mean residence time; t_1/2_, half-life; T_max_, peak time; C_max_, peak concentration.

### 3.3 CS271011 does not affect the body weight or food intake of DIO mice

**Figure 2:**
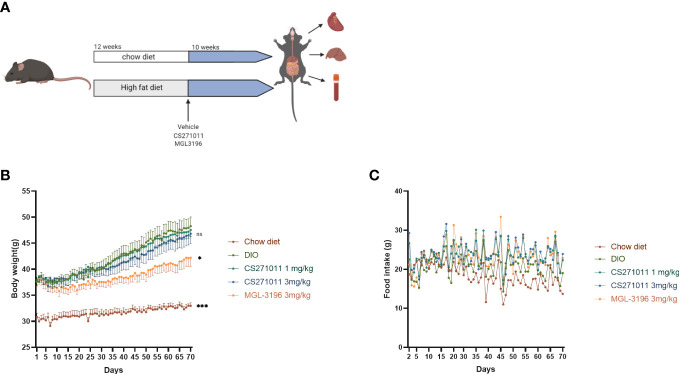
Effects of CS271011 and MGL-3196 on body weight and food intake in the DIO model. **(A)** CS271011 treatment strategy design; **(B)** Body weight change of mice during the 10-week treatment with CS271011; **(C)** Change in food intake. The results are presented as the mean ± SD. *p < 0.05 and ***p < 0.001 represent data vs. DIO controls; ns, not significant; n=39.

As illustrated in **Figure 2A**, mice were fed a chow diet or high-fat diet for 12 weeks and then received a 10-week consecutive treatment. The body weight and food intake of the animals were measured daily during treatment (**Figures 2B, C**). We observed a dramatic reduction in body weight in the MGL-3196-treated group but not in either CS271011 group (**Figure 2B**). However, neither CS271011 nor MGL-3196 impeded food intake in DIO mice (**Figure 2C**). The data suggest that CS271011 demonstrates a low impact on animal weight.

### 3.4 CS271011 lowers the levels of serum triacylglycerol and cholesterol and causes no liver injury

**Figure 3:**
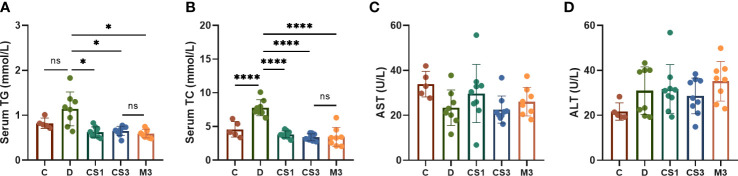
Effects of CS271011 and MGL-3196 on serum parameters in the DIO model. **(A)** Serum TG level; **(B)** Serum total TC level; **(C)** Serum AST level; **(D)** Serum ALT level. All data are expressed as the mean ± SD. *p < 0.05 and ****p < 0.0001 represent data vs. DIO controls; ns, not significant. C, chow diet control group (n=5); D, DIO control group (n=8); CS1, CS271011 1 mg/kg group (n=9); CS3, CS271011 3 mg/kg group (n=9); M3, MGL-3196 3 mg/kg group (n=8); TG, triglycerides; TC, cholesterol; AST, aspartate transaminase; ALT, alanine transaminase.

To explore the effect of CS271011 on ameliorating serum lipid content in DIO mice, serum TG and TC were measured (**Figures 3A, B**). DIO mice displayed 1.56-fold and 1.96-fold higher serum TG and TC concentrations compared with chow diet controls, indicating that the model was successfully established for further evaluation. After 10 weeks of treatment, CS271011 and MGL-3196 showed a significant reduction in serum TG and TC compared to the DIO control, whereas CS271011 dose-dependently decreased serum TC. Furthermore, we observed similar efficacy of CS271011 and MGL-3196 at the same dosage for both serum indices. We also observed that serum ALT and AST were not significantly elevated in DIO mice and the treatment group, suggesting that this model exhibited no severe hepatic injury, and no influence of CS271011 and MGL-3196 was observed on ALT and AST levels, suggesting that neither drug may cause liver damage (**Figures 3C, D**). Besides, serum ALP and TBIL level was tested for further validation of drug hepatotoxicity (**Supplementary Figure 1**), no significant change was observed in different concentrations of CS271011 group compared to control group, suggesting that CS271011 exhibited hepatic safety.

### 3.5 CS271011 alleviates liver lipid accumulation and steatosis

**Figure 4:**
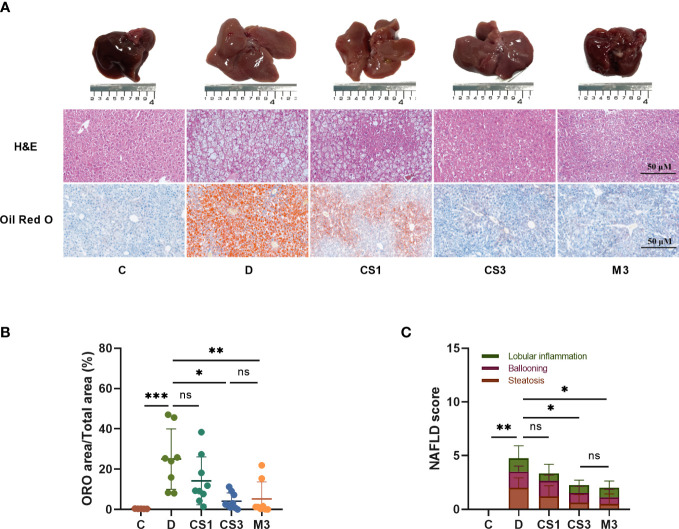
CS271011 alleviated liver steatosis and hepatic lipid accumulation. **(A)** Representative images of morphological differences in H&E staining and Oil red O staining of liver tissue among five different groups. (x20 magnification, scale bars= 50 µm); **(B)** Quantitative analysis of Oil red O staining; **(C)** NAFLD score changes in different groups of animals (C: n=5; D: n=8; CS1: n=9; CS3: n=9; M3: n=8). All data are presented as the mean ± SD. *p < 0.05, **p < 0.01, and ***p < 0.001 represent data vs. DIO controls; ns, not significant. C, chow diet control group (n=5); D, DIO control group (n=8); CS1, CS271011 1 mg/kg group (n=9); CS3, CS271011 3 mg/kg group (n=9); M3, MGL-3196 3 mg/kg group (n=8); ORO, Oil red O; NAFLD, Non-alcoholic fatty liver disease.

**Figure 5:**
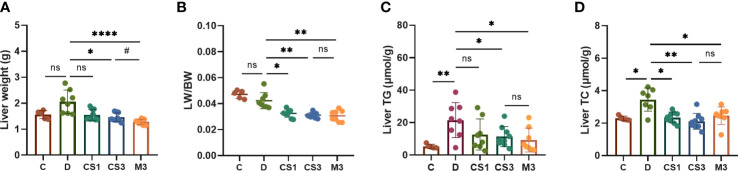
CS271011 decreased liver weight and improved hepatic biochemical indices. **(A)** Liver weight changes in the five groups. **(B)** The ratio of liver weight to body weight; **(C)** Liver TG; **(D)** Liver TC. The results are expressed as the mean ± SD, *p < 0.05, **p < 0.01, and ****p < 0.0001 represent data vs. DIO controls; #p < 0.05 represent MGL-3196 3 mg/kg group vs. CS271011 3 mg/kg group; ns, not significant. C, chow diet control group (n=5); D, DIO control group (n=8); CS1, CS271011 1 mg/kg group (n=9); CS3, CS271011 3 mg/kg group (n=9); M3, MGL-3196 3 mg/kg group (n=8); LW, liver weight; BW, body weight.

After sacrifice, the mice were weighed, and the livers and hearts were removed and weighed to calculate the ratio of liver weight to body weight (LW/BW). As expected, DIO mice exhibited a larger liver volume (**Figure 4A**) and higher liver weight (**Figure 5A**) than those of chow diet controls. The LW/BW between control and model mice was not significantly different due to the higher increase in body weight of DIO mice (**Figure 5B**). After a 10-week drug intervention, both CS271011 and MGL-3196 dramatically reduced liver weight and LW/BW, in which CS271011 showed a dose-dependent and comparative effect with MGL-3196 at the same dose (**Figure 5A**). Moreover, CS271011 and MGL-3196 did not influence heart weight or the ratio of heart weight to body weight (HW/BW), suggesting minor adverse effects on the heart for both drugs (**Supplementary Figure S2)**.

As the liver is responsible for lipogenesis and is mainly targeted by CS271011, the pathological changes in DIO mice, whether they were reverted by the agonist, are the gold standard for dyslipidaemia or NAFLD; thus, we qualified liver indices such as steatosis and ballooning by H&E and ORO staining (**Figure 4A**). The results showed that both CS271011 and MGL-3196 at the same dose (3 mg/kg) decreased the ORO staining area (**Figure 4B**) and steatosis score (**Figure 4C**), while 1 mg/kg CS271011 showed no significant effect in either assay. Both drugs at a dose of 3 mg/kg also mitigated hepatic ballooning with a considerable effect but not lobular inflammation (**Figure 4C**), while CS271011 1 mg/kg displayed no significant mitigation for either index. The average NAS score for the DIO model was approximately 5, which meets the criteria for mild NASH. CS271011 at 3 mg/kg but not 1 mg/kg dramatically reduced the NAS score in DIO mice with equivalent efficacy to that of the MGL-3196-treated group.

To confirm the role of CS271011 in hepatic lipid metabolism, we also performed assays to determine liver TG and TC levels (**Figures 5C, D**). As expected, CS271011 3 mg/kg along with MGL-3196 markedly reduced both hepatic TG and TC concentrations, while CS271011 1 mg/kg only significantly lowered liver TC. Together, at the same dose, CS271011 and MGL-3196 share comparative pathological effects on the liver in DIO mice.

### 3.6 CS271011 strongly regulates lipid metabolism-related gene expression and primarily impacts the cholesterol and steroid metabolism pathways

**Figure 6:**
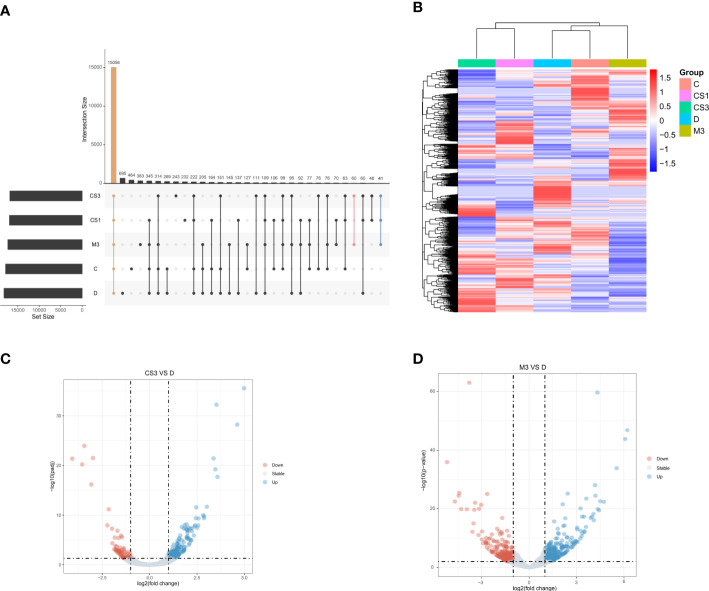
Hepatic gene expression of the different groups assessed by RNA sequencing (n=3 for each group). **(A)** The UpSet plot shows the distribution of DEGs in the five groups. The vertical histogram represents the total number of genes in different events, and the left bar plot represents the size of the gene set in the five groups. The dots represent different groups, and the dotted line shows the groups involved in the event. Orange indicates the largest number of the same genes in all five groups. Pink indicates the largest number of the same genes in the CS3 and M3 groups. Blue indicates the group with the largest number of the same genes included in the CS1, CS3, and M3 groups; **(B)** Heatmap of DEGs between groups; **(C)** Volcano plot of DEGs between groups (CS3 vs. D); **(D)** Volcano plot of DEGs between groups (M3 vs. D). D, DIO control group; CS3, CS271011 3 mg/kg group; M3, MGL-3196 3 mg/kg group; DEGs, differentially expressed genes.

**Figure 7:**
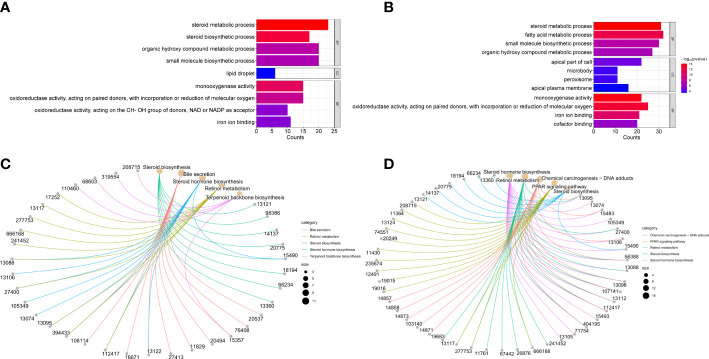
GO and KEGG enrichment analysis of hepatic DEGs in the CS271011 3 mg/kg group and MGL-3196 3 mg/kg group. **(A)** GO classification of DEGs (CS3 vs. D); **(B)** GO classification of DEGs (M3 vs. D); **(C)** KEGG classification of DEGs (CS3 vs. D); **(D)** KEGG classification of DEGs (M3 vs. D). D, DIO control group; CS3, CS271011 3 mg/kg group; M3, MGL-3196 3 mg/kg group; GO, Gene Ontology; KEGG, Kyoto Encyclopedia of Genes and Genomes; BP, biological process; CC, cellular component; MF, molecular function.

**Figure 8:**
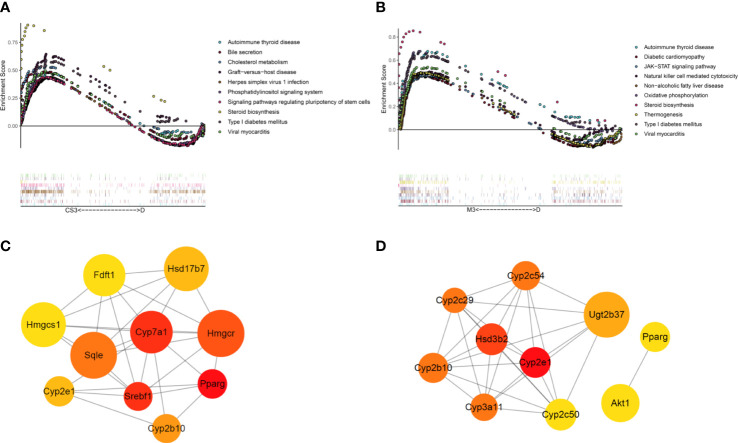
GSEA and PPI network of DEGs in the CS271011 3 mg/kg group and MGL-3196 3 mg/kg group. **(A)** GSEA of DEGs (CS3 vs. D); **(B)** GSEA of DEGs (M3 vs. D); **(C)** Nodes of the PPI network of screened DEGs and hub genes (CS3 vs. D); **(D)** Nodes of the PPI network of screened DEGs and the top ten hub genes (M3 vs. D). Nodes represent different proteins, the color of the nodes ranges from red to yellow, is related to the degree of interaction, and the size of a node is positively correlated with the log2FoldChange value. D, DIO control group; CS3, CS271011 3 mg/kg group; M3, MGL-3196 3 mg/kg group; GSEA, Gene Set Enrichment Analysis; PPI, protein−protein interaction.

To further explore the mechanism of CS271011 in lipid metabolism, we assessed the differentially expressed genes (DEGs) of the liver in different drug-treated groups by RNA-sequence analysis, and the distribution of DEGs in five groups is shown in the UpSet plot (**Figure 6A**). The standard for significance differences of DEG screening included an adjusted *P* value (padj) < 0.05 and log2FoldChange > 1. Compared with chow diet controls, DIO mice showed 55 upregulated and 35 downregulated genes, most of which were intimately associated with lipid localization, lipid transport, and metabolism pathways (**Supplementary Figures S3A, C** and **Figure S4A**). Compared with the DIO control group, mice treated with CS271011 1 mg/kg (**Supplementary Figures S3B, D** and **Figure S4B**), CS271011 3 mg/kg (**Figure 6C**), and MGL-3196 3 mg/kg (**Figure 6D**) showed significant changes in 142, 220 and 456 genes, respectively. GO and KEGG analyses showed that DEGs in the CS271011 3 mg/kg group were primarily enriched in steroid metabolic process, bile secretion, and cholesterol metabolism (**Figures 7A, C**). In comparison, the enrichment of significant DEGs in the MGL-3196 treatment group was mainly in the fatty acid metabolic process, steroid metabolism, and PPAR signaling pathway (**Figures 7B, D**). Gene Set Enrichment Analysis (GSEA) revealed the enriched DEGs in both groups. DEGs in the CS271011 3 mg/kg group were primarily enriched in steroid biosynthesis, the phosphatidylinositol signaling system, and cholesterol metabolism (**Figure 8A**), while the results in the MGL-3196 treatment group were primarily enriched in steroid biosynthesis, oxidative phosphorylation, and autoimmune thyroid disease (**Figure 8B**). In conclusion, the results of the difference in pathways between CS271011 and MGL-3196 group showed that CS271011 might prefer impact the metabolism of steroid and cholesterol, while MGL-3196 also impacted the fatty acid metabolism except for influencing the same pathway of CS271011. The results of GSEA in the DIO model and CS271011 1 mg/kg group are shown in **Supplementary Figures S4C, D**.

Then, the PPI network was applied to verify the top 10 cluster genes upon treatments, most of which in the CS271011 3 mg/kg group were involved in lipid and steroid metabolism, such as 3-hydroxy-3-methylglutaryl-coenzyme A reductase (*Hmgcr*), cholesterol 7α hydroxylase (*Cyp7a1*) and squalene epoxidase (*Sqle*), in which the first two genes encode rate-limiting enzymes for cholesterol and bile acid synthesis, and the latter one functions in steroid biosynthesis (**Figure 8C**). Interestingly, MGL-3196 diversely regulated these pathways but through different genes, such as cytochrome P450, family 2, subfamily e, polypeptide 1 (*Cyp2e1*) and cytochrome P450, family 3, subfamily a, polypeptide 11 (*Cyp3a11*) in lipid metabolism and hydroxy-delta-5-steroid dehydrogenase, 3 beta- and steroid delta-isomerase 2 (*Hsd3b2*) in steroid function (**Figure 8D**). The hub genes of the DIO and CS271011 1 mg/kg groups analyzed by the PPI network are shown in **Supplementary Figures S4E, F**.

**Figure 9:**
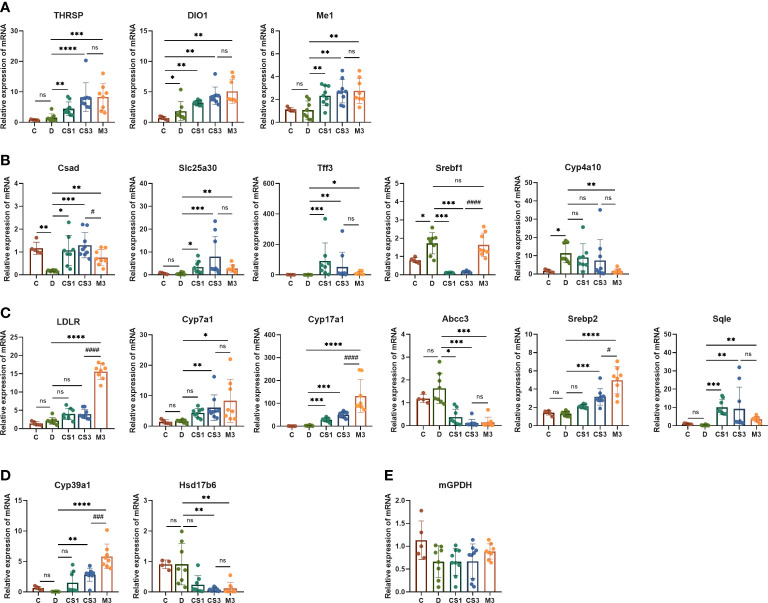
The expression of THR, hepatic and cardiac genes related to lipid and energy metabolism was validated by RT−PCR. **(A)** THR related genes; **(B)** Lipid metabolism-related genes; **(C)** Cholesterol metabolism-related genes; **(D)** Steroid metabolism-related genes; **(E)** Myocardial energy metabolism-related genes. All data are presented as the mean ± SD. *p < 0.05, **p < 0.01, ***p < 0.001, and ****p < 0.0001 represent data vs. DIO controls; #p < 0.05, ###p < 0.001, and ####p < 0.0001 represent MGL-3196 3 mg/kg group vs. CS271011 3 mg/kg group; ns, not significant. C, chow diet control group (n=5); D, DIO control group (n=8); CS1, CS271011 1 mg/kg group (n=9); CS3, CS271011 3 mg/kg group (n=9); M3, MGL-3196 3 mg/kg group (n=8).

Subsequently, we validated these dye-regulated genes by RT−PCR (**Figure 9**). Genes such as cysteine sulfinic acid decarboxylase (*Csad*), solute carrier family 25 member 30 (*Slc25a30*), and trefoil factor family peptide 3 (*Tff3*), which were downregulated in DIO mice, were rescued by both CS271011 and MGL-3196. In contrast, lipogenesis genes such as sterol regulatory element binding transcription factor 1 (*Srebf1*) was strongly downregulated by both agonists, and cytochrome P450, family 4, subfamily a, polypeptide 10 (*Cyp4a10*) was obviously downregulated by MGL-3196, limiting hepatic lipid accumulation. Furthermore, both CS271011 and MGL-3196 positively regulated the expression of genes involved in cholesterol-related metabolism, such as low-density lipoprotein receptor *(LDLR*), *Cyp7a1*, cytochrome P450 family 17 subfamily A member 1 (*Cyp17a1*), sterol regulatory element binding transcription factor 2 (*Srebp2*), and *Sqle*, and negatively regulated ATP binding cassette subfamily C member 3 (*Abcc3*). In addition, in the regulation of steroid metabolism, both agonists upregulated the expression of cytochrome P450 family 39 subfamily A member 1 (*Cyp39a1*) and diminished the expression of hydroxysteroid 17-beta dehydrogenase 6 (*Hsd17b6*). Furthermore, the THR-related genes, such as thyroid hormone responsive (*Thrsp*), iodothyronine deiodinase 1 (*Dio1*), and malic enzyme 1 (*Me1*), were also tested for the validation of THR activation.

### 3.7 CS271011 caused no effect on heart and kidney

The RNA-seq of the heart indicated that both dosages of CS271011 caused no significant DEGs of the heart compared to the DIO model (**Supplementary Figure S5**), and the expression of genes associated with myocardial energy metabolism, like mitochondrial glycerol 3-phosphate dehydrogenase (*mGPDH*) was validated by RT-PCR, showing no significant changes among all groups (**Figure 9E**). Furthermore, in the kidney cell line (HMC and NRK49F), both CS271011 and MGL-3196 exhibited no effect on cell viability, demonstrating that both compounds might be safe in kidney cells (**Supplementary Figure S6**).

## 4 Discussion

An optimum THR-β agonist should display robust THR-β potency associated with metabolic regulation and minimal thyroid hormone receptor α activity, which is responsible for the main adverse effects on bone and heart. Therefore, THR-β selectivity plays a crucial role in drug development, where MGL-3196, a phase III compound, has the potential to become a key player in treating hyperlipidaemia and liver-related diseases, such as NASH (17). Whether a candidate exists that could compete with MGL-3196 to possibly address this unmet clinical need for metabolic disorders is a question that researchers are seeking to answer.

To achieve this goal, we designed a new molecular structure and synthesized a novel compound (CS271011) to verify its efficacy in a preclinical study along with MGL-3196. Our results reveal that CS271011 triggered thyroid hormone receptor β with lower AC50 and higher selectivity than MGL-3196 *in vitro*, prompting further studies on pharmacokinetics. In the pharmacokinetic study, CS271011 exhibited favourable PK properties in normal C57BL/6J mice, suggesting its potential for efficacy studies *in vivo*. Interestingly, but expectedly, CS271011 also shows liver-targeted, a critical property for treating metabolic disorders in which the liver plays a pivotal role in lipogenesis, lipid transport, and lipolysis.

Animal model selection is critical for metabolic research to recapitulate the biological and pathological mechanisms of human diseases (19). Many animal models are used to study metabolic disorders, but few of them recapitulate disease occurrence and progression in humans (20). For example, acute murine models fed only a Western diet display elevated serum TG and/or TC but without pronounced liver inflammation and fibrosis, which differs from human diseases, which are long-term consequences of an unhealthy lifestyle (21, 22). Gene modification models such as db/db or ob/ob mice share some features with human diseases, but patients with these genetic alterations only account for a small proportion (23). Accordingly, we established a DIO mouse model with a long-term high-fat diet feeding period resembling patients with metabolic disorders who have no restriction on calorie intake and exhibit complications such as obesity, diabetes, dyslipidaemia, and NAFLD. As mentioned, the DIO model displayed increased body weight, elevated serum and liver TG and TC, and higher NASs compared with chow diet controls (**Figures 2** **–** **5**). Notably, serum TG is increased in DIO mice but is still in the normal physiological range, suggesting that it is not an appropriate model to evaluate hyperlipidaemia. This model is also characterized as a mild NASH model in terms of NAS scores (~5), in which steatosis scores are the highest, then ballooning and last lobular inflammation scores. No obvious liver inflammation or fibrosis was observed in this model. This result followed the evidence that most of the high-fat diet-induced mouse models with dieting times shorter than 20-30 weeks did not exhibit significant liver inflammation and fibrosis (22). Furthermore, this DIO model was applied to evaluate the efficacy of most THR-β agonists, such as MGL-3196 and VK2809, which are in phase II or III clinical trials (12, 18, 24).

Therefore, we hypothesize that CS271011 probably competes with MGL-3196 in the DIO mouse study based on its activity and PK study, having comparative effects with MGL-3196 *in vivo* (as the PK study showed) but with higher activation of THR-β *in vitro*. As expected, the animal study demonstrates comparative efficacy between these two agonists under the same dose in all tested parameters. However, a low dose of CS271011 (1 mg/kg) does not show competitive results with MGL-3196 (3 mg/kg), which may be due to the inadequate PK properties of CS271011, which has even greater THR-β selectivity. Generally, the only difference is that MGL-3196 but not CS27011 controls weight gain in DIO mice. We then analyzed the clinical data of MGL-3196 in NASH patients and found that diarrhoea is one of the most severe side effects reported (16). However, other THR-β agonists, such as VK2809, do not exhibit weight reduction effects in the same murine model (12, 18, 24), indicating that CS271011 may be safer in this respect.

To further discover the safety profile of CS271011, we also compared heart weights and HW/BW among different groups (**Supplementary Figure S2**), which are associated with heart-related diseases, such as hyperthyroidism. As shown, both CS271011 and MGL-3196 are proven to be safe for the heart as a result of their low THR-α activity. *mGPDH* is an indicator of the adverse effect on the heart, which is induced by T3 (25), whereas CS271011 along with MGL-3196 barely affects its expression in the hearts of DIO mice (**Figure 9E**). Furthermore, RNA-seq data of the heart in the different groups showed that almost no significant DEGs existed in the CS271011 group compared to the DIO model (**Supplementary Figure S5**), indicating that CS271011 caused no adverse effect on the heart. In the test of kidney cell lines, neither CS271011 nor MGL-3196 did not performed cytotoxicity, further confirming that CS271011 had liver-specific effect without other tissue damage (**Supplementary Figure S6**). For hepatoxicity, no elevation of AST, ALT, ALP and TBIL level was observed in CS271011 treatment group, demonstrating that this compound was safety and caused no liver injury (**Figures 3C, D** and **Supplementary Figure S1**).

Mechanistically, CS271011 also shares a similar gene-regulation panel with MGL-3196 in DIO mice (**Figures 6** **–** **8**). Further analysis revealed that CS271011 may differentiate gene regulation in cholesterol and sterol metabolism, but more research needs to be conducted.

## 5 Conclusion

We developed a novel oral liver-targeted THR-β agonist, CS271011, with high potency, selectivity, and potential to treat metabolic diseases such as hypercholesterolemia and NAFLD. However, the off-target effect on the heart and other organs and the amelioration of hepatic inflammation and fibrosis by CS271011 need further investigation in NASH and liver fibrosis models.

## Data availability statement

The original contributions presented in the study are publicly available. This data can be found here: https://www.ncbi.nlm.nih.gov the SRA accession numbers SRR22463062-SRR22463091, BioSample accessions SAMN31936366-SAMN31936395 and BioProject accession PRJNA906730.

## Ethics statement

The animal study was reviewed and approved by Shenzhen Peking University - The Hong Kong University of Science and Technology Medical Center’s Ethical Committee for the Welfare of Laboratory Animals (approval no. 2022-988).

## Author contributions

SLin performed the design and conduct of the animal experiment, data collection and analysis, and daft-writing. SH participated in the design of the study and daft-writing. The compound was designed and synthesized by ZD. YZhang participated in the design of an animal experiment. LH, YW, ZW, NH, and LW were involved in conducting the animal experiment and analysis. SLv, ZC, GY, and WY finished the data correction and analysis. YZhou and ZF conceived this study, gave funding support, and participated in revising the manuscript. All authors read and approved the final manuscript.
