# Supplementary material for: Discovery of a novel, liver-targeted thyroid hormone receptor-β agonist, CS271011, in the treatment of lipid metabolism disorders
Source: Front Endocrinol (Lausanne). 2023 Jan 20;14:1109615. doi: 10.3389/fendo.2023.1109615 (PMC9896003; doi:10.3389/fendo.2023.1109615)
Supplement: Supplementary file 1 [file DataSheet_1.docx]

**Supplementary Code**

**Code of GO analysis plot**

install.packages("colorspace")

install.packages("stringi")

install.packages("ggplot2")

install.packages("digest")

install.packages("GOplot")

if (!requireNamespace("BiocManager", quietly = TRUE))

install.packages("BiocManager")

BiocManager::install("org.Hs.eg.db")

BiocManager::install("DOSE")

BiocManager::install("clusterProfiler")

BiocManager::install("enrichplot")

library("clusterProfiler")

library("org.Hs.eg.db")

library("enrichplot")

library("ggplot2")

library(GOplot)

pvalueFilter=0.05

qvalueFilter=0.05

colorSel="qvalue"

if(qvalueFilter>0.05){

colorSel="pvalue"

}

setwd("C:\\biowolf\\fatty\\37.GO")

rt=read.table("riskDiff.txt", header=T, sep="\t", check.names=F)

genes=as.vector(rt[,1])

entrezIDs=mget(genes, org.Hs.egSYMBOL2EG, ifnotfound=NA)

entrezIDs=as.character(entrezIDs)

gene=entrezIDs[entrezIDs!="NA"]

#gene=gsub("c\\(\"(\\d+)\".*", "\\1", gene)

kk=enrichGO(gene=gene,OrgDb=org.Hs.eg.db, pvalueCutoff=1, qvalueCutoff=1, ont="all", readable =T)

GO=as.data.frame(kk)

GO=GO[(GO$pvalue<pvalueFilter & GO$qvalue<qvalueFilter),]

write.table(GO,file="GO.txt",sep="\t",quote=F,row.names = F)

showNum=10

if(nrow(GO)<30){

showNum=nrow(GO)

}

pdf(file="barplot.pdf", width=8, height=7)

bar=barplot(kk, drop = TRUE, showCategory =showNum,split="ONTOLOGY",label_format=50, color = colorSel) + facet_grid(ONTOLOGY~., scale='free')

print(bar)

dev.off()

pdf(file="bubble.pdf", width=8, height=7)

bub=dotplot(kk,showCategory = showNum, orderBy = "GeneRatio",split="ONTOLOGY", label_format=50, color = colorSel) + facet_grid(ONTOLOGY~., scale='free')

print(bub)

dev.off()

go=data.frame(Category=GO$ONTOLOGY, ID=GO$ID, Term=GO$Description, Genes = gsub("/", ", ", GO$geneID), adj_pval = GO$p.adjust)

genelist <- data.frame(ID = rt$gene, logFC = rt$logFC)

row.names(genelist)=genelist[,1]

circ <- circle_dat(go, genelist)

termNum =8

termNum=ifelse(nrow(go)<termNum,nrow(go),termNum)

geneNum=300

geneNum=ifelse(nrow(genelist)<geneNum, nrow(genelist), geneNum)

chord <- chord_dat(circ, genelist[1:geneNum,], go$Term[1:termNum])

pdf(file="GOcircos.pdf", width=11, height=11)

GOChord(chord,

space = 0.001,

gene.order = 'logFC',

gene.space = 0.25,

gene.size = 5,

border.size = 0.1,

process.label = 6)

dev.off()

**Code of KEGG analysis plot**

library(clusterProfiler)

library("org.Mm.eg.db")

library(ggplot2)

library(enrichplot)

setwd()

fr<-read.csv()

gene<-bitr(fr$Genes,fromType = 'SYMBOL',toType = 'ENTREZID',OrgDb = 'org.Mm.eg.db')

KEGG<-enrichKEGG(

gene$ENTREZID,

organism = "mmu",

keyType = "kegg",

pvalueCutoff = 0.05,

pAdjustMethod = "BH",

minGSSize = 5,

maxGSSize = 500,

qvalueCutoff = 0.2,

use_internal_data = FALSE)

enrichplot::cnetplot(KEGG,circular=TRUE,colorEdge = TRUE)

**Code of GSEA plot**

install.packages("ggplot2")

library(plyr)

library(ggplot2)

library(grid)

library(gridExtra)

setwd("")

files=grep(".xls",dir(),value=T)

data = lapply(files,read.delim)

names(data) = files

dataSet = ldply(data, data.frame)

dataSet$pathway = gsub(".xls","",dataSet$.id)

gseaCol=c("#58CDD9","#7A142C","#5D90BA","#431A3D","#91612D","#6E568C","#E0367A","#D8D155","#64495D","#7CC767","#223D6C","#D20A13","#FFD121","#088247","#11AA4D")

pGsea=ggplot(dataSet,aes(x=RANK.IN.GENE.LIST,y=RUNNING.ES,fill=pathway,group=pathway))+

geom_point(shape=21) + scale_fill_manual(values = gseaCol[1:nrow(dataSet)]) +

labs(x = "", y = "Enrichment Score", title = "") + scale_x_continuous(expand = c(0, 0)) +

scale_y_continuous(expand = c(0, 0),limits =c(min(dataSet$RUNNING.ES-0.02), max(dataSet$RUNNING.ES+0.02))) +

theme_bw() + theme(panel.grid =element_blank()) + theme(panel.border = element_blank()) +

theme(axis.line = element_line(colour = "black")) + theme(axis.line.x = element_blank(),axis.ticks.x = element_blank(),axis.text.x = element_blank()) +

geom_hline(yintercept = 0) + guides(fill=guide_legend(title = NULL)) +

theme(legend.background = element_blank()) + theme(legend.key = element_blank())

pGene=ggplot(dataSet,aes(RANK.IN.GENE.LIST,pathway,colour=pathway))+geom_tile()+

scale_color_manual(values = gseaCol[1:nrow(dataSet)]) +

labs(x = "Mutation<--------------->wild type", y = "", title = "") +

scale_x_discrete(expand = c(0, 0)) + scale_y_discrete(expand = c(0, 0)) +

theme_bw() + theme(panel.grid = element_blank()) + theme(panel.border = element_blank()) + theme(axis.line = element_line(colour = "black"))+

theme(axis.line.y = element_blank(),axis.ticks.y = element_blank(),axis.text.y = element_blank())+ guides(color=FALSE)

gGsea = ggplot_gtable(ggplot_build(pGsea))

gGene = ggplot_gtable(ggplot_build(pGene))

maxWidth = grid::unit.pmax(gGsea$widths, gGene$widths)

gGsea$widths = as.list(maxWidth)

gGene$widths = as.list(maxWidth)

dev.off()

pdf('multipleGSEA apical.pdf',

width=9,

height=5)

par(mar=c(5,5,2,5))

grid.arrange(arrangeGrob(gGsea,gGene,nrow=2,heights=c(.8,.3)))

dev.off()

**Code of volcano plot**

library(ggplot2)

dataset <- read.csv("",header = T)

cut_off_P.value = 0.01

cut_off_logFC = 1

dataset$change=ifelse(dataset$P.Value<cut_off_P.Value&abs(dataset$logFC)>=cut_off_logFC,ifelse(dataset$logFC>cut_off_logFC,"Up","Down"),"Stable")

ggplot(dataset,aes(x=logFC,y=-log10(P.Value),colour=change))+geom_point(alpha=0.4,size=3.5)+scale_color_manual(values=c("#D6604D", "#d2dae2","#4393C3"))+geom_vline(xintercept = c(-1,1),lty=4,col="black",lwd=0.8)+geom_hline(yintercept = -log10(cut_off_P.Value),lty=4,col="black",lwd=0.8)+labs(x="log2(fold change)",y="-log10(p-value)",title="NASH_HCC VS HCV_HCC")+theme_bw()+theme(plot.title = element_text(hjust = 0.5),legend.position = "right",legend.title = element_blank())

filter_up<-subset(dataset,P.Value<0.05&logFC>1)

filter_down<-subset(dataset,P.Value<0.05&logFC< -1)

print(paste('gene_up ', nrow(filter_up)))

print(paste('gene_down ', nrow(filter_down)))

write.table(filter_up, file="example_filter_up_gene.txt", quote = F)

write.table(filter_down, file="example_filter_down_gene.txt", quote = F)
