# Supplementary material for: Discovery of a novel, liver-targeted thyroid hormone receptor-β agonist, CS271011, in the treatment of lipid metabolism disorders
Source: Front Endocrinol (Lausanne). 2023 Jan 20;14:1109615. doi: 10.3389/fendo.2023.1109615 (PMC9896003; doi:10.3389/fendo.2023.1109615)
Supplement: Supplementary file 2 [file DataSheet_2.docx]

**Supplementary Figure S1. Effect of CS271011 and MGL-3196 on serum ALP and TBIL level in DIO model.** (A) Serum ALP level in five groups; (B) Serum TBIL level. All data were presented as mean ± SD. C, chow diet control group; D, DIO control group; CS1, CS271011 1 mg/kg group; CS3, CS271011 3 mg/kg group; M3, MGL-3196 3 mg/kg group; ALP, alkaline phosphatase; TBIL, total bilirubin.

**Supplementary Figure S2.** **Effect of CS271011 and MGL-3196 on heart weight in DIO model.** (A) Heart weight change in five groups; (B) The ratio of heart weight to body weight. All data were presented as mean ± SD. C, chow diet control group; D, DIO control group; CS1, CS271011 1 mg/kg group; CS3, CS271011 3 mg/kg group; M3, MGL-3196 3 mg/kg group; HW, heart weight; BW, body weight.

**Supplementary Figure S3. Volcano plot and GO enrichment analysis of hepatic DEGs in DIO and CS271011 1 mg/kg group (n=3 each group).** (A) Volcano plot of DEGs between groups (D vs. C); (B) Volcano plot of DEGs between groups (CS1 vs. D); (C) GO classification of DEGs (D vs. C); (D) GO classification of DEGs (CS1 vs. D). C, chow diet control group; D, DIO control group; CS1, CS271011 1 mg/kg group; DEGs, Differentially Expressed Genes; GO, Gene Ontology; BP, biological process; CC, cellular component; MF, molecular function.

**Supplementary Figure S4. KEGG, GSEA enrichment analysis, and PPI network of hepatic DEGs in DIO and CS271011 1 mg/kg group (n=3 each group).** (A) KEGG classification of DEGs (D vs. C); (B) KEGG classification of DEGs (CS1 vs. D); (C) GSEA of DEGs (D vs. C); (D) GSEA of DEGs (CS1 vs. D); (E) Nodes of PPI network of DEGs and hub genes screening (D vs. C); (F) Nodes of PPI network of DEGs and the top ten hub genes screening (CS1 vs. D). Nodes represented different proteins, and the color of nodes ranged from red to yellow, was related to the degree of interaction, and the size of a node was positively correlated with the log2FoldChange value. KEGG, Kyoto Encyclopedia of Genes and Genomes; GSEA, Gene Set Enrichment Analysis; PPI, protein-protein interaction.

**Supplementary Figure S5. Cardiac gene expression of the different groups assessed by RNA sequencing (n=3 for each group).** (A) Heatmap of DEGs between groups; (B) Volcano plot of DEGs between groups (D vs. C); (C) Volcano plot of DEGs between groups (CS1 vs. D); (D) Volcano plot of DEGs between groups (CS3 vs. D); (E) Volcano plot of DEGs between groups (M3 vs. D). C, chow diet control group; D, DIO control group; CS1, CS271011 1 mg/kg group; CS3, CS271011 3 mg/kg group; M3, MGL-3196 3 mg/kg group; DEGs, differentially expressed genes.

**Supplementary Figure S6. Effect of CS271011 and MGL-3196 on cell viability in kidney cell lines.** (A) Cell viability of HMC cell; (B) Cell viability of NRK-49F cell.
