# Supplementary figures and images for: Discovery of a novel, liver-targeted thyroid hormone receptor-β agonist, CS271011, in the treatment of lipid metabolism disorders
Source: Front Endocrinol (Lausanne). 2023 Jan 20;14:1109615. doi: 10.3389/fendo.2023.1109615 (PMC9896003; doi:10.3389/fendo.2023.1109615)

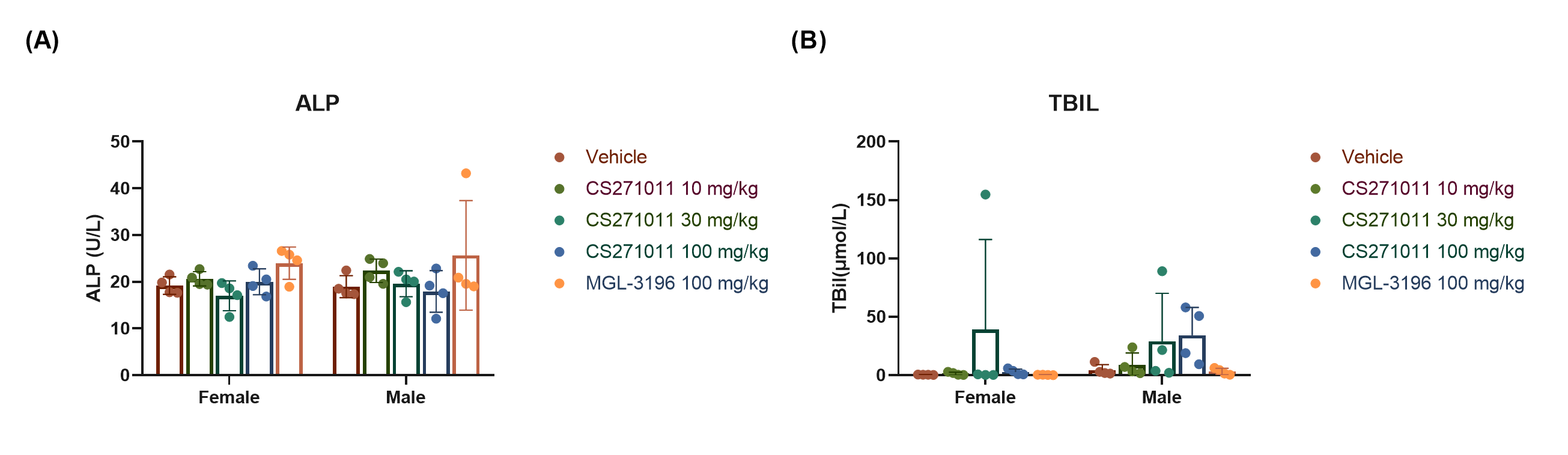

Supplement: Supplementary file 3 [file Image_1.tif]

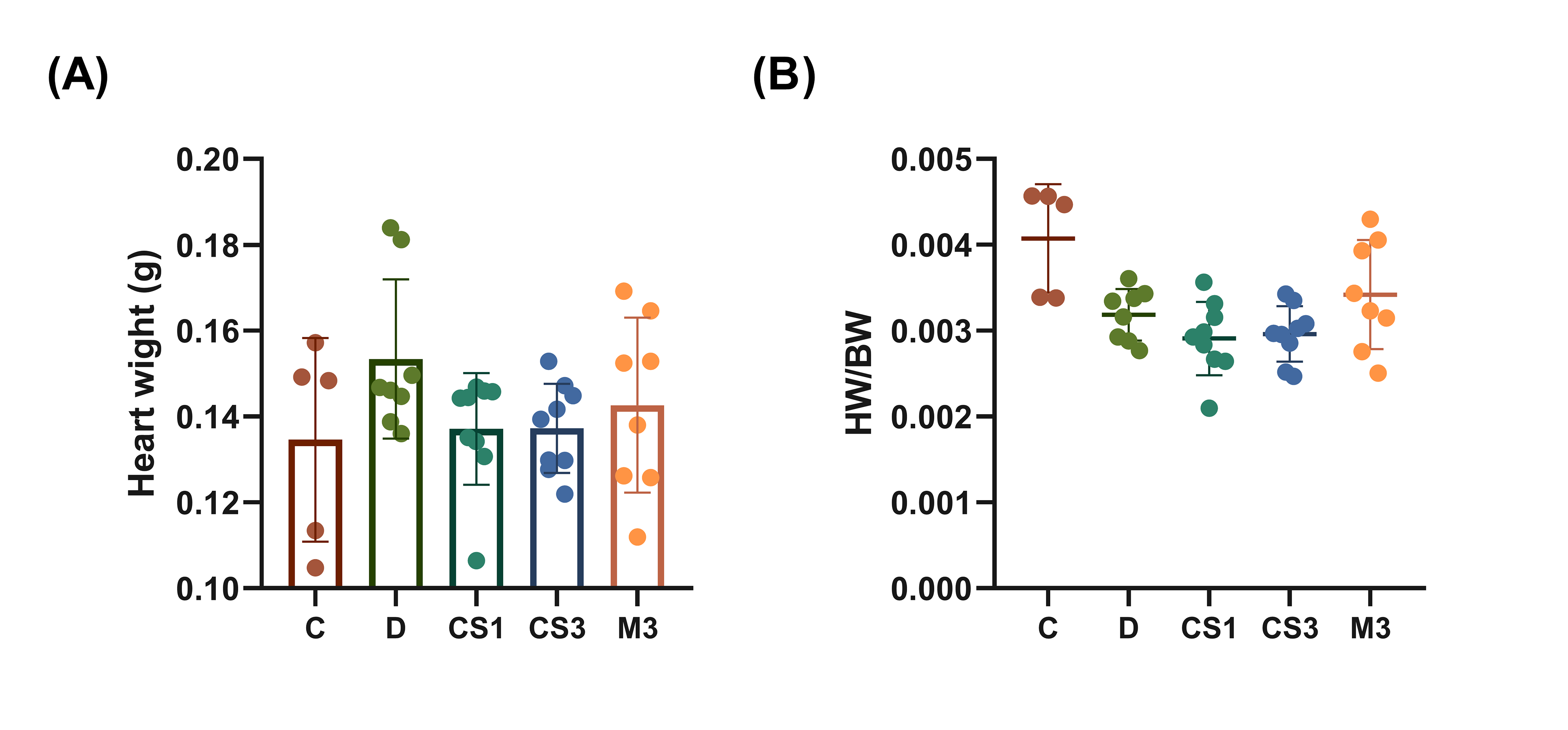

Supplement: Supplementary file 4 [file Image_2.tif]

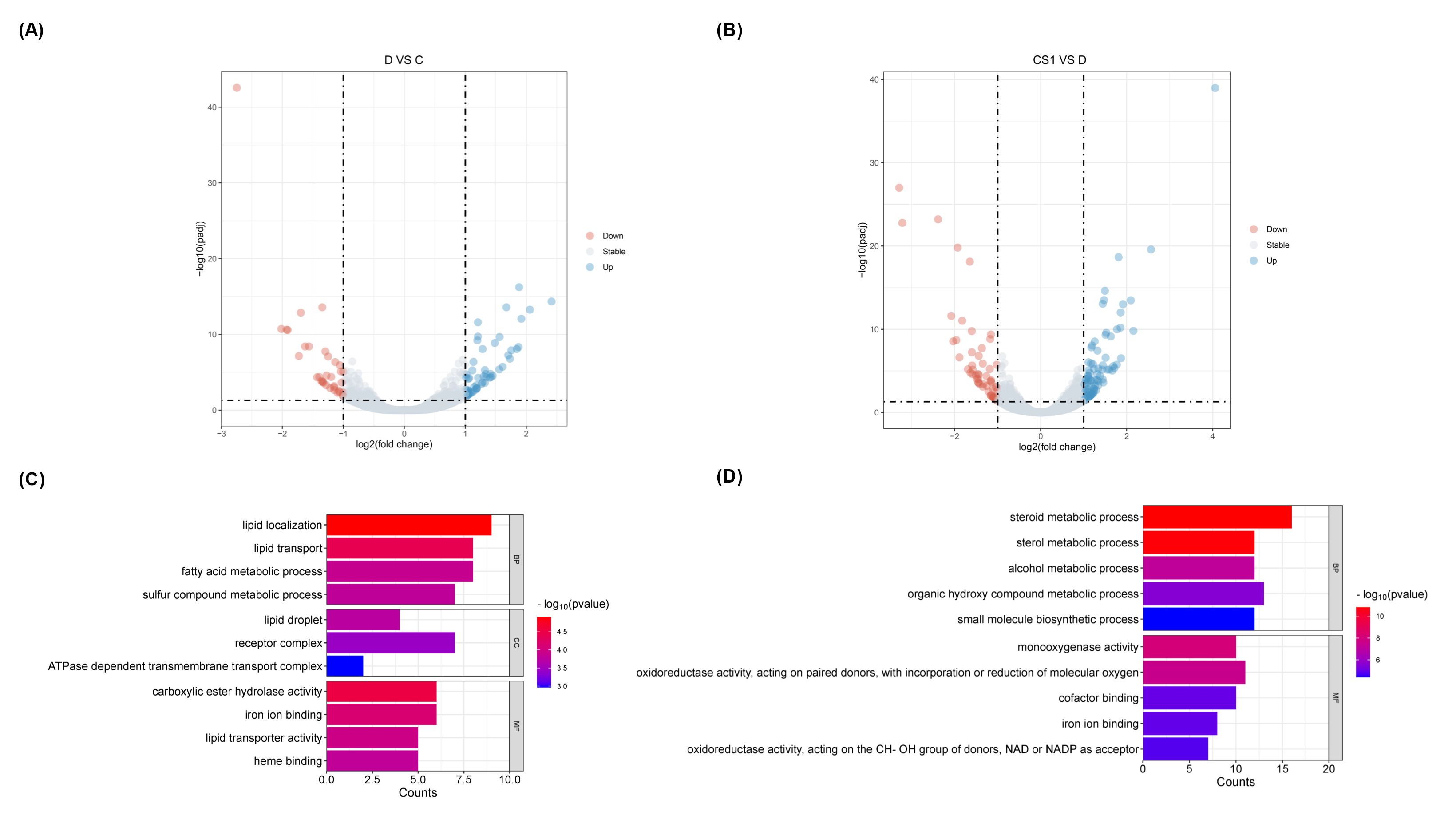

Supplement: Supplementary file 5 [file Image_3.tif]

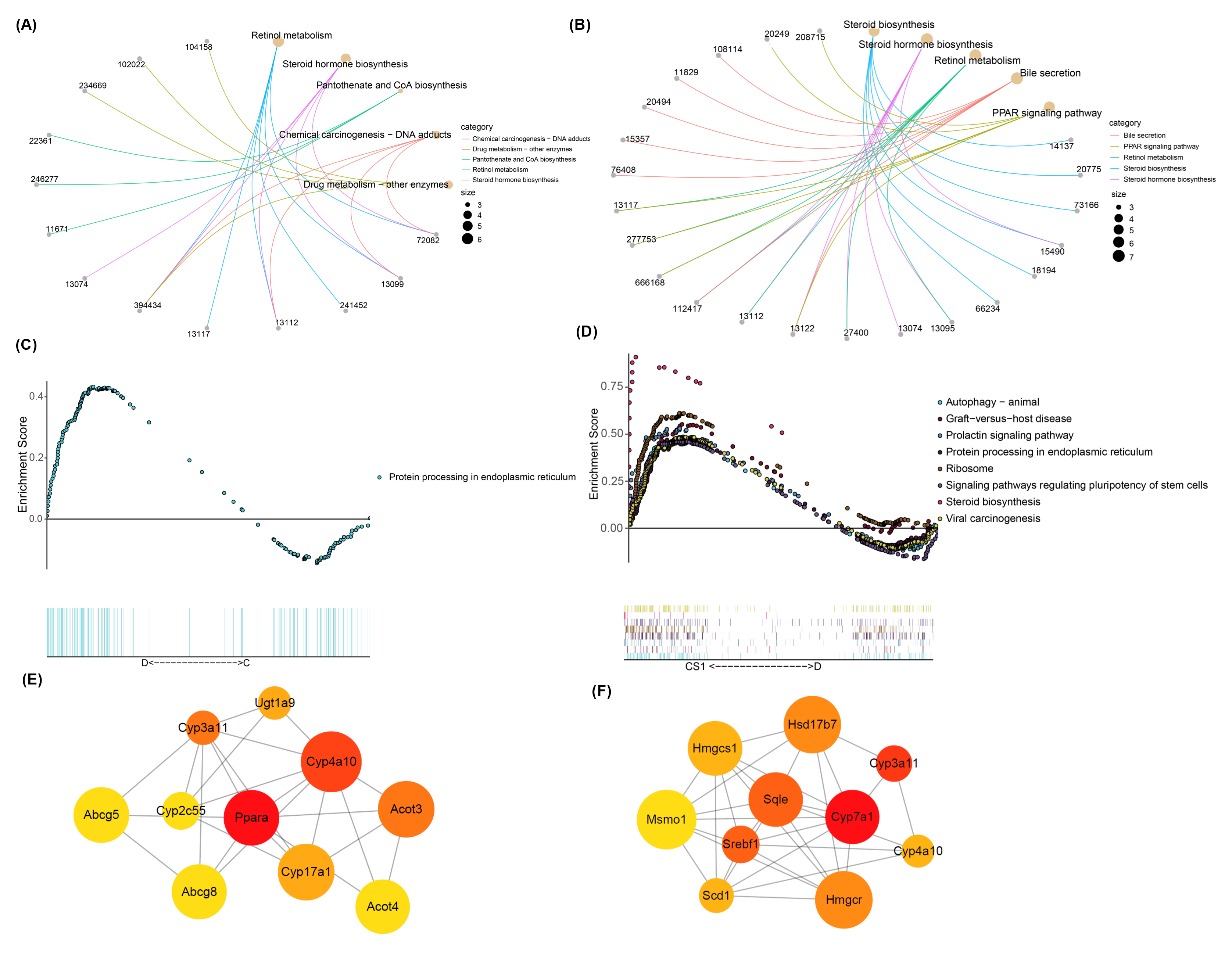

Supplement: Supplementary file 6 [file Image_4.tif]

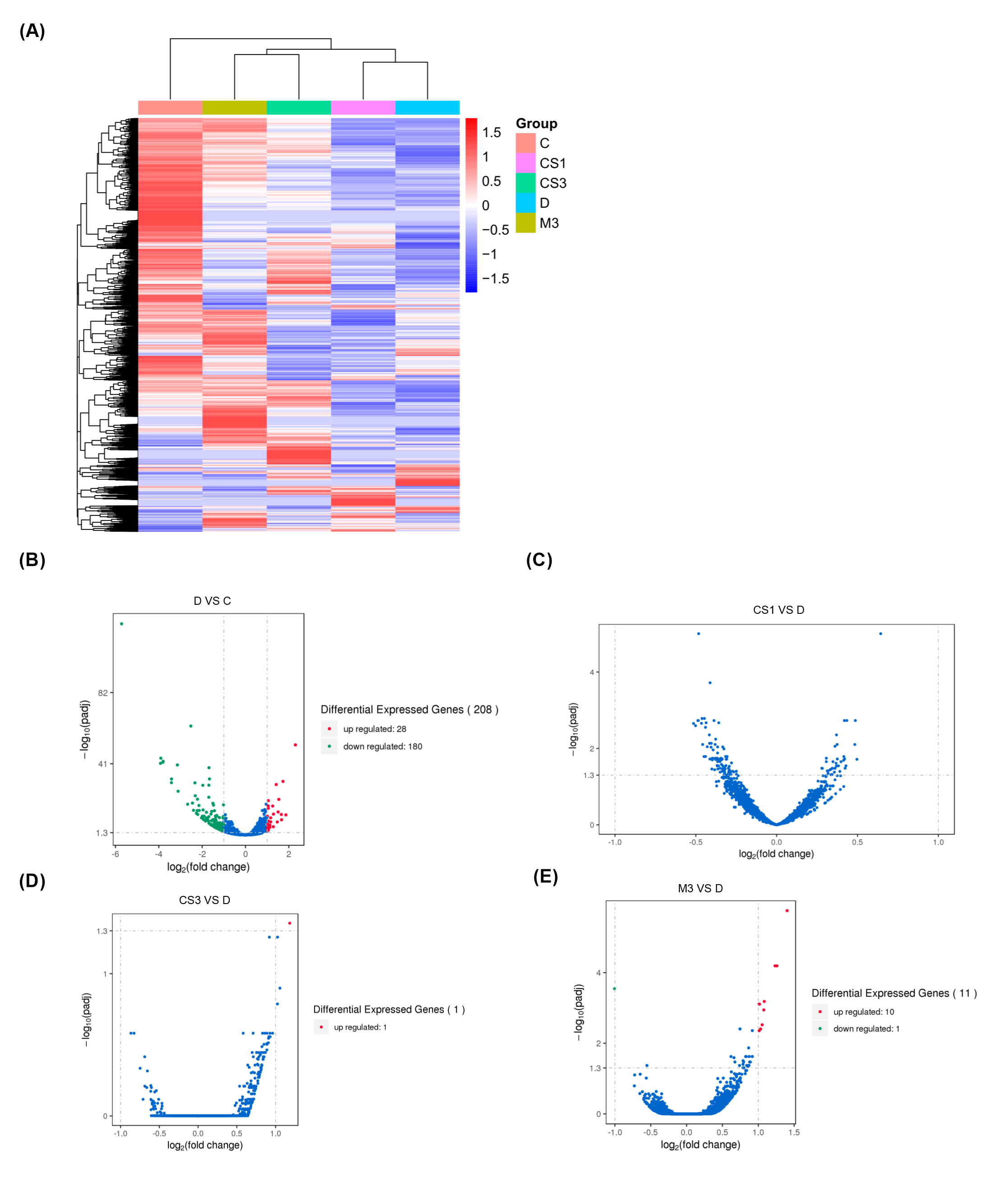

Supplement: Supplementary file 7 [file Image_5.tif]

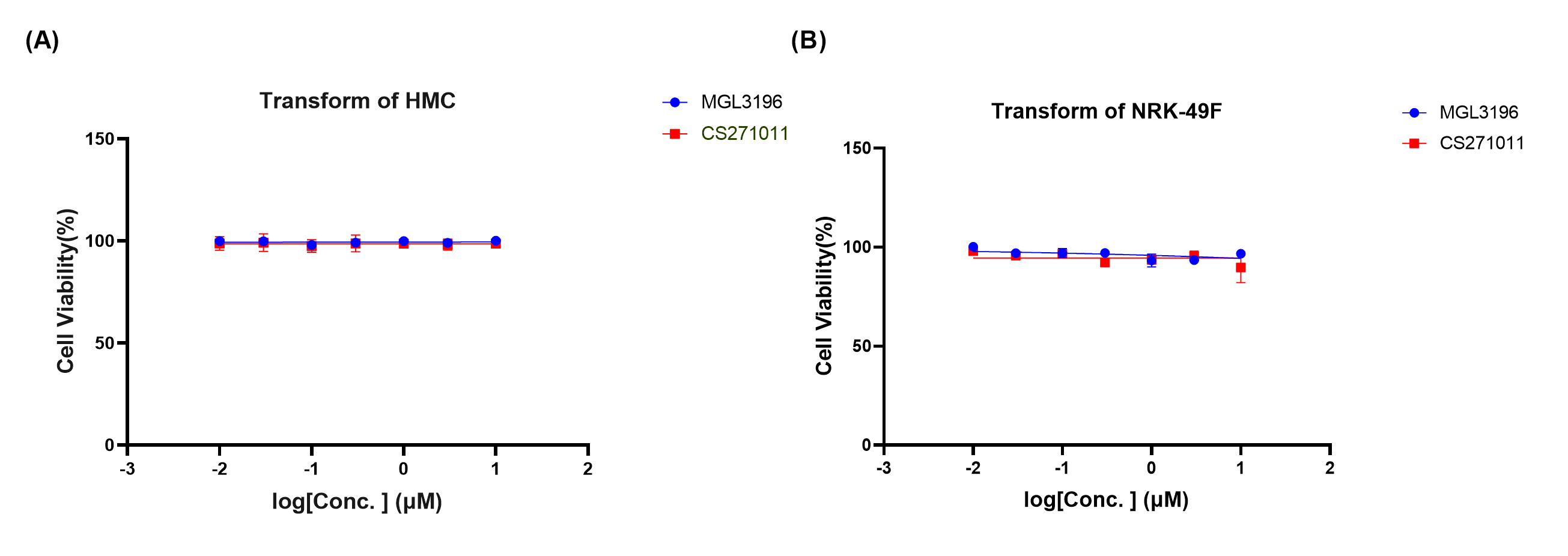

Supplement: Supplementary file 8 [file Image_6.tif]
