# Supplementary material for: Discovery of a novel, liver-targeted thyroid hormone receptor-β agonist, CS271011, in the treatment of lipid metabolism disorders
Source: Front Endocrinol (Lausanne). 2023 Jan 20;14:1109615. doi: 10.3389/fendo.2023.1109615 (PMC9896003; doi:10.3389/fendo.2023.1109615)
Supplement: Supplementary file 9 [file Table_1.docx]

**Supplementary Table 1 Primer**

| Slc25a30(M)-F: GGATTGCCTATTGCAGACATGG  Slc25a30(M)-R: GACCAAGGCGCAACCAATTT |
| --- |
| Csad(M)-F: ACACCGGAGACAAAGTGGTG  Csad(M)-R: GACGAACTCGGGCTCCATTA |
| Anxa2(M)-F: ATGTCTACTGTCCACGAAATCCT  Anxa2(M)-R: TGACTGACCCGTAGGCACTT |
| LDLR(M)-F: CCCAATGGTGGTTGCCAGTA  LDLR(M)-R: CCTGGGTGGTCAGTACAGTG |
| Cyp4a10(M)-F: AGAACTTCCCAAGTGCCTTTC  Cyp4a10(M)-R: GCAAACCATACCCATTAGCCTTT |
| Cyp17a1(M)-F: CAATGACCGGACTCACCTCC  Cyp17a1(M)-R: GGCAAACTCTCCAATGCTGG |
| Cyp7a1(M)-F: TCTACCCAGACCCTTTGACTTT  Cyp7a1(M)-R: GCTCCTGATCCAAAGGGCAT |
| Cyp39a1(M)-F: CTACTTCATGGCATTTGGAGGCG  Cyp39a1(M)-R: GGTAATGGGTCCAGAAGACTGC |
| Hsd17b6(M)-F: GTTTGACCCAGGTGACTATAAGC  Hsd17b6(M)-R: ACTTGGAGCAACTGTAGAATCCT |
| Srebf1(M)-F: CACTTCTGGAGACATCGCAAAC  Srebf1(M)-R: ATGGTAGACAACAGCCGCATC |
| Abcc3(M)-F: ACTCTCATGTGGCGAAGCAT  Abcc3(M)-R: TTACCAGCACCCGAGTCTTGC |
| Tff3(M)-F: AATGCTGTTGGTGGTCCTGG  Tff3(M)-R: CCATACATTGGCTTGGAGACAG |
| Cxcl1(M)-F: TCTCCGTTACTTGGGGACAC  Cxcl1(M)-R: CCACACTCAAGAATGGTCGC |
| Thrsp (M)-F: ATGCAAGTGCTAACGAAACGC  Thrsp (M)-R: GGAGTACCGATCCATGACTGTC |
| DIO1 (M)-F: TGGAAGACAGGGCTGAGTTTG  DIO1 (M)-R: TGCCAAAGTTCAACACCAGG |
| Me1 (M)-F: TTCATGGATTGTTGCCGCCC  Me1 (M)-R: ATCAGACATGAGCACGCTGTA |
| β-actin(M)-F: GTGACGTTGACATCCGTAAAGA  β-actin(M)-R: GCCGGACTCATCGTACTCC |
