# Supplementary material for: Discovery of a novel, liver-targeted thyroid hormone receptor-β agonist, CS271011, in the treatment of lipid metabolism disorders
Source: Front Endocrinol (Lausanne). 2023 Jan 20;14:1109615. doi: 10.3389/fendo.2023.1109615 (PMC9896003; doi:10.3389/fendo.2023.1109615)
Supplement: Supplementary file 10 [file Table_2.docx]

| Time |  | 2h | | | | 4h | | | | 24h | | | |
| --- | --- | --- | --- | --- | --- | --- | --- | --- | --- | --- | --- | --- | --- |
| Tissue |  | Blood | Heart | Liver | Kidney | Blood | Heart | Liver | Kidney | Blood | Heart | Liver | Kidney |
| Concentration  (ng/g) | **Mean**  **SD** | 1128.43  186.27 | 211.40  154.79 | 5840.63  828.57 | 979.63  340.28 | 563.64  160.94 | NA  NA | 2484.82  639.07 | 327.32  71.35 | 6.09 5.26  5.03 NA | | NA  NA | 352.03  NA |
| Ratio (tissue/blood) |  |  | 18.73% | 517.59% | 86.81% |  | NA | 440.86% | 58.07% | 86.39% | | NA | 5780.87% |

**Supplementary Table 2.1 Tissue distribution of CS271011 3 mg/kg**

| Time |  | 2h | | | | 4h | | | | 24h | | | |
| --- | --- | --- | --- | --- | --- | --- | --- | --- | --- | --- | --- | --- | --- |
| Tissue |  | Blood | Heart | Liver | Kidney | Blood | Heart | Liver | Kidney | Blood | Heart | Liver | Kidney |
| Concentration  (ng/g) | **Mean**  **SD** | 1142.53  194.69 | 243.29  45.75 | 7530.33  1551.22 | 731.74  174.20 | 910.88 69.34 | 132.62  83.85 | 1352.95  639.07 | 562.36  41.12 | 25.40 78.08  11.52 74.78 | | 80.40 45.62 | 31.71  36.82 |
| Ratio (tissue/blood) |  |  | 21.29% | 659.09% | 64.05% |  | 14.56% | 617.30% | 61.74% | 307.38% | | 316.51% | 124.84% |

**Supplementary Table 2.2 Tissue distribution of MGL-3196 3 mg/kg**

| Time |  | 2h | | | | 4h | | | | 24h | | | |
| --- | --- | --- | --- | --- | --- | --- | --- | --- | --- | --- | --- | --- | --- |
| Tissue |  | Blood | Heart | Liver | Kidney | Blood | Heart | Liver | Kidney | Blood | Heart | Liver | Kidney |
| Concentration  (ng/g) | **Mean**  **SD** | 4805.781263.17 | 743.93  400.49 | 18437.33888.61 | 3684.57  1410.64 | 2016.90 580.87 | 126.35  96.66 | 8922.73  1972.94 | 1971.69817.81 | 18.45 NA  NA NA | | NA  NA | NA  NA |
| Ratio (tissue/blood) |  |  | 15.48% | 383.65% | 76.67% |  | 6.26% | 442.40% | 97.76% | NA | | NA | NA |

**Supplementary Table 2.3 Tissue distribution of CS271011 10 mg/kg**

| Time |  | 2h | | | | 4h | | | | 24h | | | |
| --- | --- | --- | --- | --- | --- | --- | --- | --- | --- | --- | --- | --- | --- |
| Tissue |  | Blood | Heart | Liver | Kidney | Blood | Heart | Liver | Kidney | Blood | Heart | Liver | Kidney |
| Concentration  (ng/g) | **Mean**  **SD** | 4872.71 510.81 | 551.58  239.09 | 22368.62 3381.64 | 2785.89 859.95 | 3926.54 1285.19 | 439.47  152.52 | 18195.71  5935.44 | 2381.53  704.29 | 42.40 147.91  11.98 174.29 | | 203.67 49.77 | 31.11  16.81 |
| Ratio (tissue/blood) |  |  | 11.32% | 459.06% | 57.17% |  | 11.19% | 463.40% | 60.65% | 348.86% | | 480.38% | 73.37% |

**Supplementary Table 2.4 Tissue distribution of MGL-3196 10 mg/kg**

**,**
