# Supplementary material for: Discovery of a novel, liver-targeted thyroid hormone receptor-β agonist, CS271011, in the treatment of lipid metabolism disorders
Source: Front Endocrinol (Lausanne). 2023 Jan 20;14:1109615. doi: 10.3389/fendo.2023.1109615 (PMC9896003; doi:10.3389/fendo.2023.1109615)
Supplement: Supplementary file 11 [file Table_3.docx]

| Group | C | D | | CS1 | CS3 | M3 |
| --- | --- | --- | --- | --- | --- | --- |
| TC (mmol/L) | 4.58±1.23 | 7.79±1.67 | 3.83±0.54 | | 3.39±0.59 | 3.46±1.61 |
| TG (mmol/L) | 0.81±0.16 | 1.25±0.38 | 0.63±0.17 | | 0.65±0.11 | 0.59±0.11 |

**Supplementary Table 3. Serum TG and TC level.** All data are presented as the mean ± SD. C, chow diet control group (n=5); D, DIO control group (n=8); CS1, CS271011 1 mg/kg group (n=9); CS3, CS271011 3 mg/kg group (n=9); M3, MGL-3196 3 mg/kg group (n=8).
